# Executive Functions and Conceptual Models: A First Look at a Complex Topic: Commentary

**DOI:** 10.2174/0117450179462316260908052738

**Published:** 2026-09-11

**Authors:** Donatella Rita Petretto

**Affiliations:** 1 Department of Education, Philosophy, Psychology, University of Cagliari, Cagliari, Italy

**Keywords:** Executive functions, Cognitive control, Self-regulation, Theoretical models, Neurodevelopmental disorders, Major neurocognitive disorders

## Abstract

**Background:**

Since the late 19th century and over the last decades, executive functions (EF) have received increasing attention in neuropsychology and clinical psychology for their influence on various domains of functioning, activities of daily living, and various psychological and mental disorders.

**Methods:**

In this commentary, we aim to describe the major theoretical models of EF, their structures, clinical implications, with a focus on some neurodevelopmental disorders and major neurocognitive disorders.

**Results:**

Two main approaches emerge: unitary models (*e.g.*, Stuss and Benson, Norman and Shallice, Lezak) view EF as an integrated self-regulatory system, while multi-component models (*e.g*., Miyake, Diamond, Zelazo) describe separable but interconnected processes such as inhibition, working memory, and cognitive flexibility. In individuals with some neurodevelopmental disorders and major neurocognitive disorders, EF may be impaired (while sometimes only some EFs are affected, in other situations, all EFs are negatively influenced).

**Discussion:**

Contemporary EF theoretical models integrate unitary and multi-component perspectives. They describe EF as coordinated yet distinguishable control processes. Looking at some clinical conditions, such as neurodevelopmental disorders and major neurocognitive disorders, both unitary and multi-component perspectives are supported: in some specific clinical conditions, specific processes and/or modules of EF may be selectively impaired. There is still an open debate on the role of EF as a transdiagnostic feature.

**Conclusion:**

Executive functions represent a fundamental domain for understanding adaptive behaviour across the lifespan. Future research should further clarify the relationships among executive subcomponents, their neural mechanisms, and their role across psychological and neurological disorders, thereby contributing to more refined theoretical models and more targeted clinical assessment and rehabilitation strategies, as well as their role as a transdiagnostic construct.

## 1 INTRODUCTION

Since the late 19th century, executive functions (EF) have received increasing attention for their influence on everyday life and on various domains of functioning (including educational, academic, and occupational contexts). In general, executive functions are described as a set of higher-order cognitive processes that enable individuals to define goals, plan and execute strategies, regulate their behavior, adapt to environmental changes, and control their emotions [1-3]. Over the years, various authors have contributed to defining and describing the EFs [4-12].

Although interest in these functions has progressively increased, some theoretical questions remain open: How many executive functions are there? Are they a unitary function or a complex set of interrelated processes? From a clinical point of view, should they be considered as a transdiagnostic construct, present across various psychological and mental disorders (such as neurodevelopmental and major or minor neurocognitive disorders), or are specific EFs involved in specific disorders?

This commentary aims to discuss some of these topics, starting from theoretical definitions and then addressing their clinical role in specific psychological and mental disorders.

### 1.1 Conceptual Models

During the 20th century, executive functions were studied starting from their neuroanatomical bases, particularly the frontal lobe, and they were referred to as “frontal lobe functions” [4, 5]. Only later did the term “executive functions” appear, and a more articulated conceptualization of executive functions was developed.

The well-known clinical case of Phineas Gage can be considered an important starting point for the study of executive functions [6]. In 1848, Phineas Gage had a serious workplace accident in which an iron rod penetrated his skull; the iron rod destroyed a large portion of his medial frontal lobe (bilaterally). He survived but, as his physician claimed, Gage “was no longer Gage”: his basic cognitive abilities remained intact, but his personality and his capacity for planning and self-control were profoundly compromised [6].

Some years later, other clinical reports were proposed. Damasio [6] described the experience of Elliott, a man who, after the surgical removal of a meningioma in the frontal region, showed preserved general cognitive abilities but a severe inability to make appropriate decisions, regulate his emotions, and organize daily life.

In the 1980s, Stuss and Benson [4] provided a detailed description of frontal functions, defining them as a set of cognitive and behavioral abilities; they also described the domains regulated by frontal mechanisms. The authors highlighted the role of frontal lobe integrity in the coordination of those cognitive and mental functions. According to these authors, the frontal lobe and the EF act as a kind of “conductor of the orchestra,” which integrates information from different cognitive systems and directs behavior toward complex goals.

In the same years, Norman and Shallice [5] developed a cognitive model of executive control, the so-called Supervisory Attentional System (SAS). They used the SAS to describe how individuals manage novel, complex, or even conflicting situations that cannot be handled through automatic behavioral routines.

Since the 1980s, Lezak has addressed executive functions and proposed an approach to be used both in clinical and rehabilitative contexts [7]. According to Lezak, executive functions comprise four fundamental processes: goal formulation, planning strategies, execution of goal-directed actions, and monitoring of results with possible correction [7].

Some years later, Miyake and colleagues [8] proposed a model of executive functions based on three core components: updating, shifting, and inhibition. Updating refers to the ability to refresh and replace information held in working memory according to task demands. Shifting concerns the capacity for cognitive flexibility, that is, the ability to switch between tasks or strategies. Inhibition refers to the suppression of automatic or dominant responses that are inappropriate to the current situation. This model has had a major impact because it enabled the study of executive functions using rigorous experimental methods, demonstrating that these components are interrelated yet separable. The authors described this mixed structure of EF, and they referred to it as the “unity and diversity model” [8, 13-16].

In the same years, similarly to Miyake and colleagues’model [8], Diamond [9, 10] developed a developmental model of executive functions; she highlighted the role of the three core executive functions (inhibition, working memory, and cognitive flexibility) during the various phases of life; moreover, she described environmental and relational/social factors that may influence EF (quality of educational experiences, social relationships, stress levels, and physical activity) [9, 10].

In the same years, Philip Zelazo [11, 12, 17, 18] described executive functions as processes that are constructed and transformed across the lifespan, in close connection with and influenced by relational experiences and educational practices. According to his framework, executive functions are deeply tied to reflective and metacognitive capacities, and he highlighted the role of emotions and social interactions in their development. He was the first to introduce the distinction between cognitive and socio-emotional dimensions, proposing the distinction between “hot” and “cool” executive functions [17]. In Zelazo’s model, “cool” executive functions refer to abstract, decontextualized cognitive processes (cognitive flexibility, working memory, and planning) that are typically activated in emotionally neutral contexts. In contrast, “hot” executive functions operate in situations with a high emotional or motivational load, involving impulse regulation, delayed gratification, and modulation of affective responses. Zelazo points out that these dimensions, while interacting, may develop at different rates, thereby influencing self-regulation, learning, and behavioral adaptation [17, 19].

Recently, Diamond defined EFs as a “*family of top-down mental processes that make it possible for us to pay attention and stay focused; reason and problem solve; exercise choice, discipline, and the self-control to avoid being impulsive, rash, or reacting without thinking; see things from different perspectives; mentally consider alternatives, see how different ideas or facts relate to one another, and reflect on the past or consider an imagined future; and flexibly adjust to change or new information*” [20].

The models described thus far highlight two main theoretical perspectives, and they can be divided into two main groups, with some overlaps. The first group proposed a unitary view of EFs: for example, Lezak’s model [7] described executive functions as an integrated cycle of self-regulation. The second group proposed a multicomponent view, as seen in the models of Miyake and colleagues [8], Diamond [9, 10], and Zelazo [17], which interpret executive functions as a set of distinct yet interconnected components. Moreover, together, all those models also reveal a distinction between process-oriented models [5]; Lezak [7]) and modular models (*e.g.*, Miyake and colleagues [8], Diamond [10], Zelazo [17]. Each of these theoretical frameworks contributes to our understanding of different facets of executive functioning: Stuss and Benson [4] emphasized the neuroanatomical dimension; Norman and Shallice [5] introduced a cognitive framework for voluntary control; Lezak [7] focused on clinical implications of executive functions; Miyake and colleagues [8] provided an experimental and empirical structure; Diamond (2013) [10] focused on development, education and intervention; and Zelazo (2012) [11] highlighted the role of emotional dimensions.

### 1.2 The Clinical Role of Executive Functions in Neurodevelopmental Disorders and Major Neurocognitive Disorders

The clinical significance of executive functions is evident not only in cases of frontal lobe lesions, but also across various psychological/mental disorders. Their clinical role is broad and multifactorial, as executive functions play a crucial role in ensuring adaptive functioning in everyday life. Their impairment may manifest as difficulties in attentional control, inhibition of automatic responses, working memory, strategic planning, and cognitive flexibility, with significant repercussions for an individual’s functioning and adaptation in learning, social, and occupational settings. In the following sections, we specifically address the role of EFs in some neurodevelopmental disorders and some major and minor neurocognitive disorders.

Some previous and recent research has shown that children with specific learning disorders (SLD) often present EFs’ impairments, primarily in working memory but also in processing speed, long-term memory, and narrative abilities [21-23]. Working memory is essential for maintaining and manipulating temporary information; it is often impaired in children with SLD, making it difficult to integrate information during reading, writing, or mathematical problem-solving [21, 22]. Similarly, reduced processing speed may limit the ability to complete complex tasks efficiently, leading to cognitive overload and frustration [21, 22]. These deficits may interfere with activities in school activities and other activities of daily living. In adolescents and adults with specific learning disorders, EF difficulties may appear in different but equally significant forms. Prospective memory, the ability to remember to perform intended actions in the future, is often impaired, with practical consequences such as forgetting appointments, deadlines, or planned activities [24, 25]. Time management, closely linked to planning and goal prioritization, may also be inefficient, leading to reduced productivity and increased stress. Working memory continues to play a critical role in adulthood, influencing the ability to retain relevant information while solving complex problems or managing multiple tasks simultaneously [24, 25]. These deficits may interfere with occupational functioning and other activities of daily living.

In older adults with major and minor neurocognitive disorders, EFs are central to maintaining autonomy and quality of life, and their decline is frequently observed in both major and minor neurocognitive disorders [26, 27]. These disorders represent a heterogeneous group of syndromes characterized by progressive cognitive decline in various domains of functioning that interferes with daily functioning and personal independence. Among the various cognitive domains affected, EFs are particularly affected, exhibiting patterns of deficit that vary depending on the specific type of neurocognitive disorder [26, 27].

In major neurocognitive disorder due to Alzheimer’s disease, initial impairment primarily affects episodic memory, yet EF difficulties - particularly in planning complex actions, cognitive flexibility, and behavioral monitoring and correction - often emerge in the early stages. These deficits interfere with everyday activities such as meal preparation, financial management, and scheduling tasks [27-32].

Frontotemporal major and minor neurocognitive disorders display a distinct cognitive profile. In these conditions, executive dysfunction represents the predominant symptom from the earliest phases of the disease. People with frontotemporal major neurocognitive disorders may exhibit impulsivity, deficient inhibitory control, rigid thinking, and an inability to adapt behavior to environmental demands [33, 34]. Such alterations reflect the involvement of prefrontal regions, which are responsible for the regulation of executive processes, and profoundly affect an individual’s social and relational functioning.

In major neurocognitive disorder due to Lewy Body disease (LBD), EFs may be affected early in the onset of the disorder [27]. Individuals with LBD may show difficulties with planning, problem solving, and cognitive flexibility, which influence their ability to adapt to changes or manage complex tasks. In individuals with LBD, attention and alertness tend to fluctuate, with periods of confusion alternating with clearer moments [35]. Impulsive responses or repetitive behaviors are common due to poor inhibitory control. Working memory is also impaired, affecting the capacity to store and manipulate information. These problems are linked to dysfunction in fronto-striatal circuits and prefrontal regions. Compared with Alzheimer’s disease, LBD typically shows greater executive dysfunction but relatively better episodic memory [27]. Executive difficulties have an important impact on the patient’s independence and daily functioning [27, 36].

The executive difficulties observed in neurodevelopmental disorders and major neurocognitive disorders described thus far highlight two main patterns: while EFs are impaired in all the described disorders, some EFs could be more severely affected that other depending on the specific disorders, with different effects during different phases of life within the same disorder. Looking at these clinical conditions, both unitary and multi-component perspectives on EFs are supported.

## 2 DISCUSSION

In recent decades, EFs have received increasing attention in neuropsychology for their importancein clinical contexts and for their influence on various life contexts (such as educational, social, and occupational domains) and on activities of daily living [1-3]. In this paper, we discussed the major theoretical models that have been developed and identified two main perspectives: one based on a unitary construct of EFs and one based on multicomponent views of EFs. Unitary models, such as the one proposed by Lezak [7], emphasize the integrative and coordinating role of executive functions. In contrast, multi-component approaches, such as those proposed by Miyake [8], Diamond [10, 12], and Zelazo [12], focus on the identification of specific subprocesses (inhibition, working memory, cognitive flexibility, and updating). Moreover, in recent decades, some authors have highlighted the need to integrate these two different perspectives into a new coherent framework: although executive functions are composed of distinct components, they share the common goal of supporting behavioral regulation and adaptive functioning. A similar debate on unitary models and multicomponent models of EFs emerges from a clinical point of view when we look at some specific neurodevelopmental disorders and major neurocognitive disorders. EFs are negatively influenced in all the discussed disorders, but specific EFs are affected in specific neurodevelopmental disorders and specific neurocognitive disorders, supporting the “unity and diversity” approach proposed by Miyake and colleagues some years ago [8]. Moreover, this scenario could support the idea that EFs could represent a transdiagnostic construct across various mental and psychological disorders [37, 38], consistent with recent dimensional approaches to psychopathology, such as HITOP and RDoC, and with recent papers on these topics [39-43]. Furthermore, this scenario could also support the development of transdiagnostic interventions aimed at directly improving specific EFs and that cut across diagnostic categories, rather than being limited to specific diagnostic categories and boundaries [38]. However, more research is needed in these fields.

## CONCLUSION

EFs represent a fundamental domain for understanding adaptive behaviour across the lifespan. Future research should further clarify the relationships among executive subcomponents, their neural mechanisms, and their role across psychological and neurological disorders, thereby contributing to more refined theoretical models and more targeted clinical assessment and rehabilitation strategies, as well as their role as a transdiagnostic construct [37, 38].

## LIST OF ABBREVIATIONS

EF: Executive Function
SLD: Specific Learning Disorders
LBD: Lewy Body Disease
SAS: Supervisory Attentional System
